# 2-[((*E*)-2-{2-[(*E*)-2-Hy­droxy­benzyl­idene]hydrazinecarbon­yl}hydrazinyl­idene)meth­yl]phenol

**DOI:** 10.1107/S1600536811053268

**Published:** 2011-12-21

**Authors:** Rahman Bikas, Parisa Mahboubi Anarjan, Seik Weng Ng, Edward R. T. Tiekink

**Affiliations:** a Young Researchers Club, Tabriz Branch, Islamic Azad University, Tabriz, Iran; b Department of Chemistry, University of Zanjan 45195-313, Zanjan, Iran; c Department of Chemistry, University of Malaya, 50603 Kuala Lumpur, Malaysia; d Chemistry Department, Faculty of Science, King Abdulaziz University, PO Box 80203 Jeddah, Saudi Arabia

## Abstract

The mol­ecule of the title compound, C_15_H_14_N_4_O_3_, is completed by the application of crystallographic twofold symmetry, with the carbonyl group lying on the rotation axis. The mol­ecule is close to planar: the greatest deviation of a torsion angle from 0° is 7.3 (2)° about the bond linking the phenol ring to the rest of the mol­ecule. An intra­molecular O—H⋯N(imine) hydrogen bond is formed in each half of the mol­ecule. The carbonyl O atom is *anti* with respect to the amine H atoms and this allows for the formation of N—H⋯O(hydrox­yl) hydrogen bonds in the crystal, which results in supra­molecular layers lying parallel to (100).

## Related literature

For the structures of related carbohydrazides, see: Bikas *et al.* (2010*a* ▶,*b* ▶).
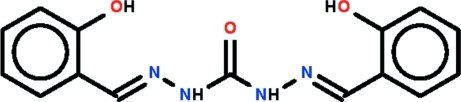


## Experimental

### 

#### Crystal data

C_15_H_14_N_4_O_3_
*M* *_r_* = 298.30
Orthorhombic, 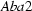

*a* = 14.3101 (4) Å
*b* = 9.3620 (2) Å
*c* = 10.2697 (2) Å
*V* = 1375.84 (6) Å^3^
*Z* = 4
Cu *K*α radiation
μ = 0.86 mm^−1^
*T* = 100 K
0.25 × 0.25 × 0.10 mm

#### Data collection

Agilent SuperNova Dual diffractometer with an Atlas detector
Absorption correction: multi-scan (*CrysAlis PRO*; Agilent, 2010 ▶) *T*_min_ = 0.342, *T*_max_ = 1.000
2323 measured reflections
757 independent reflections
750 reflections with *I* > 2σ(*I*)
*R*_int_ = 0.014

#### Refinement

*R*[*F* ^2^ > 2σ(*F* ^2^)] = 0.027
*wR*(*F* ^2^) = 0.075
*S* = 1.10
757 reflections
110 parameters
1 restraint
H atoms treated by a mixture of independent and constrained refinement
Δρ_max_ = 0.16 e Å^−3^
Δρ_min_ = −0.21 e Å^−3^

### 

Data collection: *CrysAlis PRO* (Agilent, 2010 ▶); cell refinement: *CrysAlis PRO*; data reduction: *CrysAlis PRO*; program(s) used to solve structure: *SHELXS97* (Sheldrick, 2008 ▶); program(s) used to refine structure: *SHELXL97* (Sheldrick, 2008 ▶); molecular graphics: *X-SEED* (Barbour, 2001 ▶) and *DIAMOND* (Brandenburg, 2006 ▶); software used to prepare material for publication: *publCIF* (Westrip, 2010 ▶).

## Supplementary Material

Crystal structure: contains datablock(s) global, I. DOI: 10.1107/S1600536811053268/hb6561sup1.cif

Structure factors: contains datablock(s) I. DOI: 10.1107/S1600536811053268/hb6561Isup2.hkl

Supplementary material file. DOI: 10.1107/S1600536811053268/hb6561Isup3.cml

Additional supplementary materials:  crystallographic information; 3D view; checkCIF report

## Figures and Tables

**Table 1:** Hydrogen-bond geometry (Å, °)

*D*—H⋯*A*	*D*—H	H⋯*A*	*D*⋯*A*	*D*—H⋯*A*
O2—H2⋯N2	0.86 (3)	1.79 (4)	2.5710 (17)	150 (3)
N1—H1⋯O2^i^	0.89 (3)	2.12 (3)	2.983 (2)	161 (2)

Symmetry code: (i) 
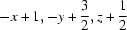
.

**Fig. 1.**
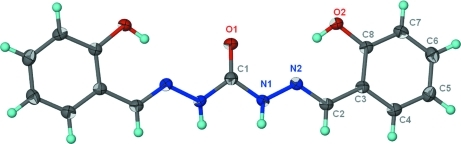
Molecular structure of (I) showing displacement ellipsoids at the 70% probability level. The molecule has twofold symmetry and the unlabelled atoms are related by the symmetry operation 1 - x, 1 - y, z.

**Fig. 2.**
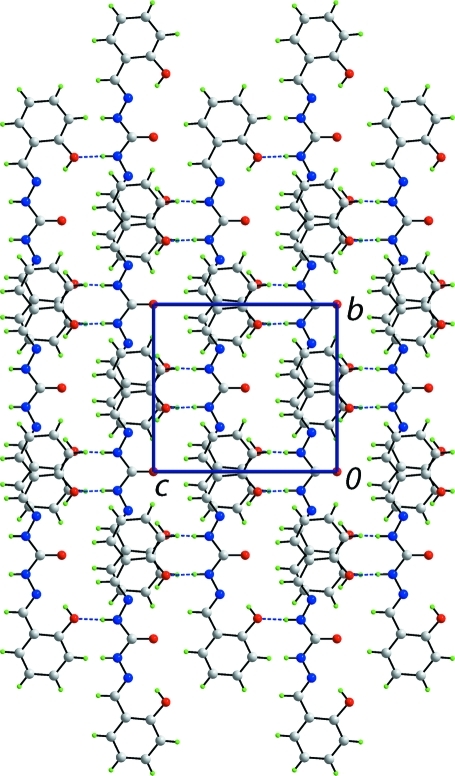
A view of the supramolecular layer parallel to (100) in (I). The N—H···O hydrogen bonds are shown as blue dashed lines.

## supplementary crystallographic information

### Comment

In connection with previous structural studies of carbohydrazide derivatives (Bikas *et al.*, 2010*a*,*b*), the title compound, (I), was investigated. The molecule, Fig. 1, has crystallographic twofold symmetry. The molecule is essentially planar with a r.m.s. deviation for all 22 atoms comprising the full molecule being 0.074 Å. The maximum deviation from 0° for a torsion angle in the molecule is 7.3 (2)° for N2—C2—C3—C8. The carbonyl-O atom is *anti* with respect to the amine-H atoms, and the conformation about the C2═N2 imine bond [1.2857 (19) Å] is *E*.The hydroxyl-H atom forms an intramolecular hydrogen bond to the imine-H atom, Table 1.

In the crystal, the amine-H atoms form hydrogen bonds to the hydroxyl-O atoms to form supramolecular layers parallel to (100), Fig. 2 and Table 1.

### Experimental

All reagents were commercially available and used as received. A methanol (10 ml) solution of 2-hydroxybenzaldehyde (3 mmol) was added drop-wise to a methanol solution (10 ml) of carbohydrazide (1.5 mmol), and the mixture was refluxed for 3 h. Then the solution was evaporated on a steam bath to 5 ml and cooled to room temperature. White precipitates of the title compound were separated and filtered off, washed with cooled methanol (3 ml) and then dried in air. Crystals of the title compound were obtained from its methanol solution by slow solvent evaporation. Yield: 94%. *M*.pt. 496–497 K. Selected IR data (cm^-1^): 3272 (v. broad, N—H), 1721 (C═O); 1625 (s, C═N(azomethine)); 959 (m, N—N); 1353, 1273 (s, C—O).

### Refinement

Carbon-bound H-atoms were placed in calculated positions [C—H = 0.95 Å, *U*_iso_(H) = 1.2 *U*_eq_(C)] and were included in the refinement in the riding model approximation. The hydroxyl and amino H-atoms were refined freely. In the absence of significant anomalous scattering effects, 242 Friedel pairs were averaged in the final refinement.

### Crystal data

**Table:**

C_15_H_14_N_4_O_3_	*F*(000) = 624
*M**_r_* = 298.30	*D*_x_ = 1.440 Mg m^−^^3^
Orthorhombic, *A**b**a*2	Cu *K*α radiation, λ = 1.54178 Å
Hall symbol: A 2 -2ac	Cell parameters from 1858 reflections
*a* = 14.3101 (4) Å	θ = 3.1–76.5°
*b* = 9.3620 (2) Å	µ = 0.86 mm^−^^1^
*c* = 10.2697 (2) Å	*T* = 100 K
*V* = 1375.84 (6) Å^3^	Prism, colourless
*Z* = 4	0.25 × 0.25 × 0.10 mm

### Data collection

**Table:**

Agilent SuperNova Dual diffractometer with an Atlas detector	757 independent reflections
Radiation source: SuperNova (Cu) X-ray Source	750 reflections with *I* > 2σ(*I*)
Mirror	*R*_int_ = 0.014
Detector resolution: 10.4041 pixels mm^-1^	θ_max_ = 76.7°, θ_min_ = 6.2°
ω scan	*h* = −17→17
Absorption correction: multi-scan (*CrysAlis PRO*; Agilent, 2010)	*k* = −11→10
*T*_min_ = 0.342, *T*_max_ = 1.000	*l* = −10→12
2323 measured reflections	

### Refinement

**Table:**

Refinement on *F*^2^	Secondary atom site location: difference Fourier map
Least-squares matrix: full	Hydrogen site location: inferred from neighbouring sites
*R*[*F*^2^ > 2σ(*F*^2^)] = 0.027	H atoms treated by a mixture of independent and constrained refinement
*wR*(*F*^2^) = 0.075	*w* = 1/[σ^2^(*F*_o_^2^) + (0.0579*P*)^2^ + 0.2173*P*] where *P* = (*F*_o_^2^ + 2*F*_c_^2^)/3
*S* = 1.10	(Δ/σ)_max_ < 0.001
757 reflections	Δρ_max_ = 0.16 e Å^−^^3^
110 parameters	Δρ_min_ = −0.21 e Å^−^^3^
1 restraint	Extinction correction: *SHELXL97* (Sheldrick, 2008), Fc^*^=kFc[1+0.001xFc^2^λ^3^/sin(2θ)]^-1/4^
Primary atom site location: structure-invariant direct methods	Extinction coefficient: 0.0051 (7)

### Special details

**Table:**

Geometry. All e.s.d.'s (except the e.s.d. in the dihedral angle between two l.s. planes) are estimated using the full covariance matrix. The cell e.s.d.'s are taken into account individually in the estimation of e.s.d.'s in distances, angles and torsion angles; correlations between e.s.d.'s in cell parameters are only used when they are defined by crystal symmetry. An approximate (isotropic) treatment of cell e.s.d.'s is used for estimating e.s.d.'s involving l.s. planes.
Refinement. Refinement of *F*^2^ against ALL reflections. The weighted *R*-factor *wR* and goodness of fit *S* are based on *F*^2^, conventional *R*-factors *R* are based on *F*, with *F* set to zero for negative *F*^2^. The threshold expression of *F*^2^ > σ(*F*^2^) is used only for calculating *R*-factors(gt) *etc*. and is not relevant to the choice of reflections for refinement. *R*-factors based on *F*^2^ are statistically about twice as large as those based on *F*, and *R*- factors based on ALL data will be even larger.

### Fractional atomic coordinates and isotropic or equivalent isotropic displacement parameters (Å^2^)

**Table:**

	*x*	*y*	*z*	*U*_iso_*/*U*_eq_	
O1	0.5000	0.5000	0.50067 (16)	0.0218 (4)	
O2	0.59665 (8)	0.87891 (12)	0.43307 (12)	0.0197 (3)	
N1	0.52439 (9)	0.61383 (13)	0.69670 (15)	0.0186 (3)	
N2	0.55917 (9)	0.73346 (13)	0.63891 (13)	0.0170 (3)	
C1	0.5000	0.5000	0.6191 (2)	0.0174 (4)	
C2	0.57681 (11)	0.84255 (16)	0.71089 (15)	0.0172 (3)	
H2A	0.5646	0.8395	0.8018	0.021*	
C3	0.61574 (10)	0.97076 (15)	0.65176 (15)	0.0159 (4)	
C4	0.64432 (11)	1.08454 (17)	0.73122 (16)	0.0191 (4)	
H4	0.6380	1.0769	0.8230	0.023*	
C5	0.68171 (11)	1.20828 (18)	0.67764 (18)	0.0211 (4)	
H5	0.7009	1.2847	0.7324	0.025*	
C6	0.69086 (11)	1.21928 (18)	0.54321 (19)	0.0219 (4)	
H6	0.7165	1.3036	0.5062	0.026*	
C7	0.66290 (11)	1.10842 (16)	0.46270 (15)	0.0195 (4)	
H7	0.6698	1.1169	0.3710	0.023*	
C8	0.62482 (9)	0.98474 (16)	0.51579 (14)	0.0159 (4)	
H1	0.5006 (17)	0.614 (2)	0.777 (3)	0.023 (5)*	
H2	0.579 (2)	0.809 (3)	0.481 (3)	0.051 (8)*	

### Atomic displacement parameters (Å^2^)

**Table:**

	*U*^11^	*U*^22^	*U*^33^	*U*^12^	*U*^13^	*U*^23^
O1	0.0307 (8)	0.0208 (7)	0.0138 (8)	−0.0031 (6)	0.000	0.000
O2	0.0260 (6)	0.0186 (5)	0.0146 (5)	−0.0020 (4)	−0.0006 (5)	−0.0009 (4)
N1	0.0253 (7)	0.0169 (7)	0.0136 (6)	−0.0027 (5)	0.0024 (5)	0.0014 (5)
N2	0.0183 (6)	0.0166 (7)	0.0160 (7)	0.0001 (5)	−0.0004 (5)	0.0014 (5)
C1	0.0181 (10)	0.0175 (10)	0.0167 (10)	0.0017 (7)	0.000	0.000
C2	0.0176 (6)	0.0190 (7)	0.0149 (7)	0.0010 (5)	−0.0001 (5)	0.0002 (6)
C3	0.0147 (7)	0.0180 (7)	0.0150 (7)	0.0022 (6)	−0.0004 (6)	0.0005 (6)
C4	0.0181 (7)	0.0219 (8)	0.0174 (8)	0.0007 (6)	−0.0004 (6)	−0.0010 (7)
C5	0.0195 (7)	0.0197 (8)	0.0241 (8)	−0.0025 (5)	0.0002 (6)	−0.0040 (6)
C6	0.0186 (8)	0.0209 (8)	0.0261 (9)	−0.0031 (5)	0.0009 (7)	0.0035 (7)
C7	0.0190 (7)	0.0230 (7)	0.0166 (8)	−0.0003 (6)	0.0009 (6)	0.0027 (6)
C8	0.0136 (7)	0.0181 (7)	0.0161 (9)	0.0016 (5)	−0.0009 (6)	−0.0003 (6)

### Geometric parameters (Å, °)

**Table:**

O1—C1	1.217 (3)	C3—C4	1.403 (2)
O2—C8	1.3660 (19)	C3—C8	1.408 (2)
O2—H2	0.86 (3)	C4—C5	1.390 (2)
N1—N2	1.3617 (16)	C4—H4	0.9500
N1—C1	1.3754 (19)	C5—C6	1.391 (3)
N1—H1	0.89 (3)	C5—H5	0.9500
N2—C2	1.2857 (19)	C6—C7	1.386 (2)
C1—N1^i^	1.3754 (19)	C6—H6	0.9500
C2—C3	1.456 (2)	C7—C8	1.391 (2)
C2—H2A	0.9500	C7—H7	0.9500
			
C8—O2—H2	106 (2)	C5—C4—H4	119.5
N2—N1—C1	118.53 (14)	C3—C4—H4	119.5
N2—N1—H1	122.6 (13)	C4—C5—C6	119.41 (17)
C1—N1—H1	116.2 (14)	C4—C5—H5	120.3
C2—N2—N1	118.33 (13)	C6—C5—H5	120.3
O1—C1—N1	125.38 (10)	C7—C6—C5	120.64 (16)
O1—C1—N1^i^	125.38 (10)	C7—C6—H6	119.7
N1—C1—N1^i^	109.2 (2)	C5—C6—H6	119.7
N2—C2—C3	119.35 (14)	C6—C7—C8	120.17 (15)
N2—C2—H2A	120.3	C6—C7—H7	119.9
C3—C2—H2A	120.3	C8—C7—H7	119.9
C4—C3—C8	118.64 (14)	O2—C8—C7	118.40 (14)
C4—C3—C2	119.67 (14)	O2—C8—C3	121.46 (14)
C8—C3—C2	121.69 (14)	C7—C8—C3	120.14 (14)
C5—C4—C3	120.99 (16)		
			
C1—N1—N2—C2	176.05 (12)	C4—C5—C6—C7	0.1 (2)
N2—N1—C1—O1	−5.97 (14)	C5—C6—C7—C8	0.3 (2)
N2—N1—C1—N1^i^	174.03 (14)	C6—C7—C8—O2	178.90 (13)
N1—N2—C2—C3	178.82 (12)	C6—C7—C8—C3	−0.9 (2)
N2—C2—C3—C4	−173.31 (13)	C4—C3—C8—O2	−178.76 (13)
N2—C2—C3—C8	7.3 (2)	C2—C3—C8—O2	0.6 (2)
C8—C3—C4—C5	−0.6 (2)	C4—C3—C8—C7	1.1 (2)
C2—C3—C4—C5	−179.99 (13)	C2—C3—C8—C7	−179.58 (13)
C3—C4—C5—C6	0.0 (2)		

Symmetry codes: (i) −*x*+1, −*y*+1, *z*.

### Hydrogen-bond geometry (Å, °)

**Table:**

*D*—H···*A*	*D*—H	H···*A*	*D*···*A*	*D*—H···*A*
O2—H2···N2	0.86 (3)	1.79 (4)	2.5710 (17)	150 (3)
N1—H1···O2^ii^	0.89 (3)	2.12 (3)	2.983 (2)	161 (2)

Symmetry codes: (ii) −*x*+1, −*y*+3/2, *z*+1/2.
